# The Association of Hospital Characteristics with Brachial Plexus Birth Injury

**DOI:** 10.21203/rs.3.rs-9032961/v1

**Published:** 2026-03-26

**Authors:** Mary Claire Manske, Barton Wise, Yueju Li, Daniel Tancredi

**Affiliations:** CHILDREN’S HOSPITAL OF PHILADELPHIA; University of California Davis; University of California Davis; University of California at Davis

## Abstract

**Objective:**

Evaluate association between hospital characteristics and brachial plexus birth injury (BPBI) and whether associations vary by race.

**Study Design:**

Retrospective study of liveborn infants from 1997–2019 using the Kids’ Inpatient Database linked to American Hospital Association Annual Survey. Multivariable logistic regression models were used to assess adjusted associations between hospital characteristics and BPBI. Interaction terms were examined to evaluate effect modification by race.

**Results:**

7,824,862 sampled infants, representing 25,400,023 infants in the population, were included. After adjustment, lower obstetric care level, complications, or hospitals within highest quartile of Black births were associated with BPBI. Associations between hospital characteristics and BPBI were consistent across racial groups.

**Conclusion:**

Hospital characteristics reflecting institutional resources, quality, and patient population are associated with BPBI. BPBI odds were greatest in hospitals with greater proportion of Black infants even after adjustment for individual sociodemographics and risk factors, suggesting additional, unmeasured structural differences in obstetric care delivery.

## Introduction

Brachial plexus birth injury (BPBI) is a traumatic injury sustained during childbirth to the cervical and thoracic nerve roots that provide motor and sensory function to the upper extremity. Occurring in 1.5 per 1000 livebirths, ^1–3^ BPBI presents as upper extremity weakness or paresis in a newborn. Although many infants recover spontaneously, up to 20% to 60% of affected infants experience permanent neurologic injury,^1,4,5^ which results in lifelong upper extremity weakness, the musculoskeletal sequelae of chronic denervation (joint contractures, skeletal dysplasia, impaired upper limb growth), and impairments in physical function and psychosocial well-being that extend into adulthood.^6^ Although numerous risk factors for BPBI have been identified, including maternal obesity, gestational diabetes, macrosomia, shoulder dystocia, prolonged labor, and vaginal delivery (compared to Cesarean section),^7,8^ prediction and prevention of these injuries remains challenging. Many of these factor are not identifiable prior to delivery, none have demonstrated sufficient predictive accuracy for clinical use,^8–12^ and up to half of affected infants have no recognized risk factors.^1^ These limitations suggest that important contributors to BPBI risk remain unidentified.

Racial and ethnic disparities in BPBI are well documented. ^2,3,13–18^ Black and Hispanic infants experience significantly higher rates of BPBI compared to White infants. Even after adjustment for known clinical risk factors, Black and Hispanic infants have 88% and 25% higher odds of BPBI, respectively, compared to White infants.^3^ Notably, these disparities do not seem to be due to a higher risk of the established BPBI risk factors, as Black and Hispanic infants have lower risk of many of the most common and strongly associated risk factors for BPBI, including shoulder dystocia, macrosomia, and vaginal birth.^19^ Consequently, the mechanisms underlying these inequities remain poorly understood.

Current research on BPBI risk factors has focused nearly exclusively on individual-level, physiologic risk factors. However, non-physiologic determinants of health at the hospital and systems levels are known to influence maternal and neonatal outcomes and contribute to racial and ethnic disparities across a range of perinatal conditions. Hospital characteristics such as delivery volume,^20–22^ obstetric care level,^23^ neonatal care level and volume,^24–26^ urban/rural location,^27,28^ teaching status,^28^ demographic composition,^28^ and hospital quality,^29,30^ have been associated with maternal and neonatal morbidity and mortality, yet their relationship to BPBI has not been examined. Because hospital characteristics are identifiable prenatally and potentially modifiable, understanding the association with BPBI may inform prevention strategies and equity-focused quality improvement initiatives. The objectives of this study were to evaluate the association between hospital characteristics and BPBI and determine whether these associations vary by race.

## Methods

After obtaining approval from the University of California Davis Institutional Review Board, we conducted a retrospective cohort study of all liveborn infants delivered in U.S. hospitals between from 1997 to 2019 using the Kids’ Inpatient Database (KID), a publicly available, nationally representative administrative database, developed as part of the Healthcare Cost and Utilization Project (HCUP).^31^ KID contains discharge-level data from a stratified sample of nonfederal, short-term hospitals, including children’s hospitals and general hospitals, and captures approximately 80% of pediatric inpatient discharges nationwide. Sampling weights were applied to allow generation of national estimates. Additionally, we obtained data from the American Hospital Association (AHA) Annual Survey of Hospitals,^32^ a nationally representative survey containing detailed information on hospital structure, services, and capabilities. For the years in which a common hospital identifier was available (2000, 2003, 2006, and 2009), we linked KID to AHA data, allowing us to collect additional characteristics of the delivery hospital from the AHA dataset.

Available infant-level variables included race/ethnicity, sex, primary payer, and median household income for the patient’s ZIP code, as well as established BPBI risk factors, including shoulder dystocia, macrosomia, mode of birth, multiple gestation, and gestational age. Race and ethnicity in KID are derived from hospital discharge records as reported by participating hospitals or state data organizations; these data reflect local collection practices (which may include patient self-report or administrative assignment). KID then standardizes these into a combined race/ethnicity variable with six categories: White, Black, Hispanic, Asian or Pacific Islander, Native American, or Other. We used this race/ethnicity variable provided by KID. Hospital characteristics included obstetric care level, annual delivery volume, ownership type, urban or rural location, teaching status, and availability of neonatal intensive or intermediate care. Obstetric care level was defined using AHA classifications, which categorize hospitals based on self-reported obstetric services and capabilities. For each hospital-year, we calculated the proportion of births among Black infants, Medicaid-insured infants, and infants from the lowest income ZIP code quartile. Hospitals in the highest quartile for each measure were classified as predominantly Black-serving, Medicaid-serving, or low-income–serving, respectively.

Hospital quality was assessed using a modified Unexpected Complications in Term Newborns quality metric derived from the California Maternal Quality Care Collaborative (CMQCC)’s Unexpected Complications in Term Newborns measure. Developed in 2011 to capture adverse perinatal outcomes associated with labor and delivery in infants expected to have healthy, uncomplicated births,^33^ this metric reflects neonatal morbidity among term infants without major congenital anomalies, based on diagnosis and procedure codes indicating respiratory, infectious, neurologic, or birth-related complications (including BPBI). In 2019, this measure was integrated into the Joint Commission’s Perinatal Care (PC) quality measures set to monitor hospital quality ^34,35^ and has been widely utilized in perinatal research.^25,29,36^ The statistical code to calculate the Unexpected Complications in Term Newborn measure was obtained from CMQCC. Our modified metric is identical to the original except that we modified the definition of the birth complications outcome by excluding BPBI to avoid endogeneity in our derived hospital-level quality metric, given our intention to use this metric as an independent variable in subsequent models with BPBI as the outcome. The Unexpected Complications in Term Newborns variable is a composite, risk-adjusted measure for each hospital for each year. The values for this variable are the predicted random intercepts for each combination of hospital and year when fitting a mixed-effects logistic regression model.

### Statistical Analysis

Descriptive statistics were calculated for infant and hospital characteristics. Multiple logistic regression models estimated adjusted associations between hospital characteristics and BPBI, controlling for infant demographics, socioeconomic factors, and established BPBI risk factors. Effect modification by race was evaluated using interactions terms between race and selected hospital characteristics, including obstetric care level and hospital demographic composition. Statistical significance was defined as p < 0.05. All statistical analysis was performed using SAS^®^ software version 9.4 for Windows^®^ (SAS Institute Inc, Cary, NC).

## Results

Our study cohort included 7,824,862 sampled infants from 9,539 hospitals weighted to a population of 25,400,023 infants in the population. Infant and hospital characteristics are summarized in Tables 1 and 2.

### Infant factors and BPBI

Associations of infant sociodemographic characteristics and known BPBI risk factors with BPBI are included in table 3. Notably, Black race and Hispanic ethnicity were associated with 62% and 29% higher odds of BPBI (aOR: 1.62, 95% CI: 1.52, 1.72, and aOR: 1.29, 95% CI: 1.22, 1.37, respectively), after controlling for other sociodemographic characteristics, known BPBI risk factors, and hospital factors. Medicaid insurance was also associated with a 14% higher odds of BPBI (aOR: 1.14, 95% CI: 1.09, 1.20).

### Hospital Characteristics and BPBI

**Figure 1:**
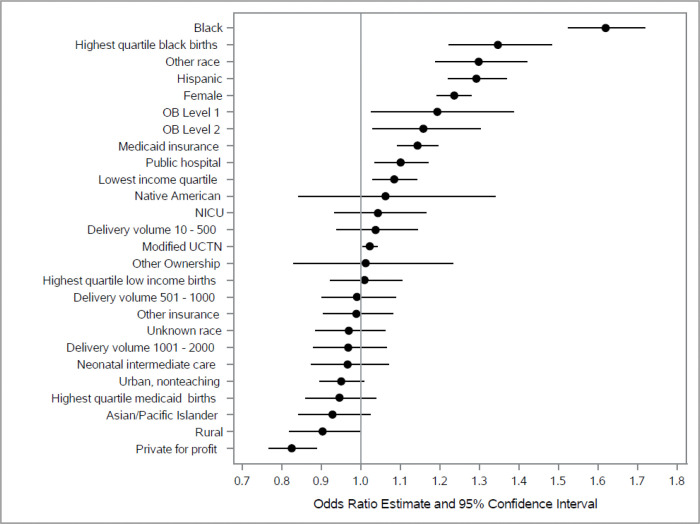


Several hospital characteristics were associated with BPBI (Table 4), including obstetric care level, location, ownership, and the modified Unexpected Complications in Term Newborns index. Compared with hospitals designated as the highest obstetric care level (level 3), infants born in level 1 and 2 hospitals had higher odds of BPBI (aOR1.19, 95% CI: 1.03, 1.39, and aOR 1.16, 95% CI: 1.03, 1.30, respectively). Infants born in rural hospitals had a 9.0% lower odds of BPBI (aOR: 0.90, 95% CI:0.82, 0.98) compared to urban teaching hospitals. Public hospital ownership was associated with a 10% higher odds of BPBI (aOR: 1.10, 95% CI:1.03, 1.17), whereas births at private- private for-profit hospitals had a 17% lower odds of BPBI (aOR: 0.83, 95% CI:0.77, 0.89). Lower hospital quality, as measured by the modified Unexpected Complications in Term Newborns metric, was associated with BPBI; for example, per interquartile range increment (e.g. the difference between hospitals in the 25^th^ and 75^th^ percentile for complications) the odds of BPBI were 3% higher (aOR: 1.031, 95% CI: 1.004, 1.059). Hospital delivery volume, presence of a neonatal intensive care unit, or neonatal intermediate care availability were not significantly associated with BPBI (Figure 1).

### Hospital Demographic Composition

Hospital demographic composition was also associated with BPBI. Infants born at hospitals in the highest quartile of Black infant births had a 35% higher odds of BPBI (aOR: 1.35, 95% CI: 1.22, 1.48), independent of individual race, insurance status, neighborhood income, and established BPBI risk factors. Hospital-level proportions of insurance, low-income ZIP code residence, and racial and ethnic composition were not significantly associated with BPBI.

### Effect modification by race

The association between obstetric care level and BPBI did not differ by race (p=0.44). Similarly, the association of delivering at a predominantly black-serving hospital (p=0.76), or predominantly low-income-serving hospital (p=0.25) with BPBI did not differ by race. However, the association between a hospital’s proportion of Medicaid-insured births differed by race (p for interaction=0.013). Among Black infants, birth in hospitals in either the second or third quartiles of Medicaid-insured births was associated with an approximately 20% lower odds of BPBI (aOR: 0.79 95% CI: 0.65, 0.95, and aOR: 0.80, 95% CI :0.66, 0.97, respectively) compared to the lowest quartile of Medicaid-insured infants, whereas no significant association was observed among non-Black infants.

## Discussion

In this population-based study of liveborn infants born in U.S. hospitals over a 22-year period, we evaluated the association between hospital characteristics and BPBI and examined whether these associations varied by race. We report three principal findings. First, several hospital characteristics, including obstetric care level and hospital quality, were independently associated with BPBI, supporting the role of systems-level factors in the occurrence of this injury. Second, the demographic composition of a hospital’s patient population was associated with BPBI risk even after adjustment for individual-level race, socioeconomic factors, and established clinical risk factors. Third, after accounting for a wide range of individual, community, and hospital-level characteristics, race and ethnicity remained associated with BPBI, underscoring the presence of unidentified drivers of persistent disparities.

### Hospital resources, capability, and quality

Our finding that higher obstetric care level was protective against BPBI aligns with a growing body of literature demonstrating that greater institutional resources and clinical capabilities are associated with better perinatal outcomes. Prior studies have shown that higher levels of maternal and neonatal care mitigate the risk of severe morbidity among high-risk patients, even after accounting for patient complexity.^22,37–41^ DeSisto and colleagues demonstrated that birth in higher-level maternal care facilities modified the relationship between maternal risk conditions and severe maternal outcomes, supporting the value of regionalized systems of care.^23^ Similarly, studies of neonatal care have consistently shown lower mortality and morbidity among very-low-birth-weight infants born at higher-level or higher-volume neonatal centers.^24,40^

Although BPBI has traditionally been conceptualized as an unpredictable obstetric complication, our findings suggest that institutional context matters. Obstetric care level may reflect not only the availability of subspecialty services, but also differences in staffing models, team experience, access to rapid consultation, and standardized approaches to managing intrapartum emergencies. These structural features may influence the recognition and management of shoulder dystocia or other high-risk delivery scenarios, thereby affecting BPBI risk even when traditional clinical risk factors are present.

In addition to care level, we found that hospital quality as measured using a modified Unexpected Complications in Term Newborns index was associated with BPBI. This metric was developed to capture severe neonatal morbidity among term infants expected to have uncomplicated births and has been widely adopted as a hospital-level quality indicator. The association between higher complication rates and BPBI risk suggests that BPBI may occur within broader patterns of intrapartum and immediate neonatal care processes, rather than representing an entirely isolated event. Together, these findings support the concept that BPBI is influenced not only by individual clinical circumstances, but also by the systems in which care is delivered.

### Hospital quality and racial and ethnic disparities

Hospital quality has also been shown to contribute meaningfully to racial and ethnic disparities in perinatal outcomes.^29,30,36^ Prior studies using the Unexpected Complications in Term Newborns metric have demonstrated higher rates of severe neonatal morbidity among Black and Hispanic infants compared with White and Asian infants, disparities that persist after adjustment for maternal sociodemographic and clinical characteristics.^29^ Importantly, Black and Hispanic infants are more likely to be born in hospitals with higher risk-adjusted complication rates, implicating birth location as a contributor to inequities in neonatal outcomes.^42^

Our findings extend this literature to BPBI. The observed association between hospital quality and BPBI suggests that disparities in exposure to lower-quality obstetric care environments may contribute to inequities in this injury. While BPBI is uncommon relative to other neonatal morbidities, its lifelong functional consequences make these disparities particularly consequential.

### Hospital patient mix and BPBI risk

A novel finding of this study is that the demographic composition of a hospital’s patient population was associated with BPBI. Infants born in hospitals with the highest proportion of births among Black individuals experienced substantially higher odds of BPBI, even after controlling for individual race, insurance status, neighborhood income, known BPBI risk factors, and multiple hospital characteristics. This suggests that the patient mix of a birth hospital conveys risk beyond individual-level sociodemographic characteristics alone.

Similar patterns have been observed for other maternal and neonatal outcomes. Prior work has demonstrated higher maternal mortality and morbidity rates in predominantly Black-serving hospitals, with disparities persisting both within and between hospital types.^28^ These findings raise the possibility that structural differences in resources, staffing, institutional support, or cumulative exposure to systemic underinvestment may affect care processes in ways not fully captured by traditional hospital-level variables.

Notably, among Black infants, birth at hospitals in the middle quartiles of Medicaid-serving hospitals was associated with lower BPBI risk compared with hospitals in the lowest quartile, suggesting that the relationship between hospital patient mix and outcomes is complex and not straightforward. These findings highlight the need for more granular investigation into how institutional context, payer mix, and resource allocation interact to influence perinatal outcomes among historically marginalized populations.

### Persistent racial and ethnic disparities

Despite extensive adjustment for clinical, socioeconomic, community-level, and hospital-level factors, race and ethnicity remained among the strongest predictors of BPBI in our models. Importantly, inclusion of hospital characteristics did not attenuate the magnitude of these disparities. This pattern mirrors prior BPBI studies demonstrating that Black and Hispanic infants remain at higher risk even though these populations are less likely to exhibit several established risk factors, such as macrosomia or shoulder dystocia.^19^

These findings suggest that race and ethnicity may function as proxies for unmeasured or inadequately captured exposures. Potential contributors include differential intrapartum management,^43^ variation in clinician response to emerging complications, implicit bias,^44,45^ communication barriers,^46^ differences in escalation of care, or cumulative effects of structural racism within healthcare systems^47,48^. Administrative data are not well suited to capture these mechanisms, yet their influence may be substantial.

Taken together, our results indicate that while systems-level characteristics meaningfully influence BPBI risk, they do not fully explain observed racial and ethnic disparities. Addressing inequities in BPBI will likely require interventions that extend beyond traditional risk stratification and encompass structural, institutional, and interpersonal dimensions of obstetric care.

### Strengths and limitations

This study has several limitations. Its retrospective design and reliance on administrative data introduce the potential for misclassification, unmeasured confounding, and incomplete capture of relevant clinical and social variables. Maternal characteristics were not available, precluding dyadic analyses that may further elucidate BPBI risk. Additionally, obstetric care level was derived from American Hospital Association self-reported data, which may overestimate hospital capabilities and does not directly measure clinical performance or outcomes. Our modified Unexpected Newborn Complications index excluded BPBI to avoid outcome contamination, which may have attenuated its association with injury risk.

Strengths include the large, nationally representative sample spanning more than two decades, linkage of multiple data sources, and simultaneous evaluation of individual-, community-, and hospital-level factors. This comprehensive approach allowed us to examine BPBI within a broader systems-of-care framework not previously explored.

### Implications

In conclusion, we found that BPBI may be influenced by institutional context and healthcare systems. Strategies such as risk-appropriate care, regionalization, and targeted quality improvement efforts may represent important opportunities for prevention. However, the persistence of racial and ethnic disparities despite extensive adjustment underscores the need for future research focused on structural drivers of inequity, including hospital-level practices, care processes, and broader manifestations of structural racism within perinatal care.

## Figures and Tables

**Table 1:** Demographic characteristics of the study cohort

Infant Characteristics				
Total live births, weighted n	25,400,023			
	Unweighted count	Unweighted percentage	Weighted count	Weighted Percentage
**Female**	3,345,929	46.8	12,367,574	48.7
**Race/Ethnicity**				
White	3,082,325	43.1	1,117,9805	44.0
Black	959,532	13.4	2,938,759	11.6
Hispanic	1,269,671	17.7	4,541,181	17.9
Asian/Pacific Islander	317,474	4.4	1,071,678	4.2
Native American	41,626	0.6	13,924	0.5
Other	397,626	5.6	1,339,122	5.3
**Payer**				
Private insurance	3,451,924	48.2	12,700,852	50.1
Medicaid	3,149,897	44	10,670,747	42.1
Other	540,843	7.6	1,976,318	7.8
**Lowest-income quartile**	1,781,341	24.9	6,061,460	24.2
**BPBI**	19,207	0.3	28,081	0.11
**Intrapartum Factors**				
Shoulder dystocia	40,144	0.6	83,557	0.3
Macrosomia (>4.5kg)	111,001	1.6	404,950	1.6
Cesarean	2,697,908	37.7	7,857,070	30.9
Multiple gestation	435,084	6.1	823,818	3.2
>41 weeks gestational age	616,821	8.6	1,928,506	7.6

**Table 2:** Hospital Characteristics

Weighted birth level distribution of Hospital Characteristics	
Hospital Characteristics	Unweighted N (weighted %)
**Delivery volume (annual)**	
10–500	2,063,310 (35)
501–1000	2,101,723 (29)
1001–2000	2,070,537 (26)
>2000	917,252 (10)
**Teaching status**	
Rural	619,087 (12)
Urban Non-teaching	2,371,082 (36)
Urban teaching	4,122,942 (52)
**Ownership**	
Public (Government, nonfederal)	2,617,690 (41)
Private, not-for-profit	3,562,332 (44)
Private, for profit	812,847 (12)
Other	120,242 (3)
**Obstetric care level (2000–2009)**	
Level 1	370,679 (16)
Level 2	707,888 (30)
Level 3	860,108 (36)
Missing	428,985 (18)
**Neonatal ICU (2000–2009)**	
Yes	1,357,841 (57)
No	717,373 (30)
Missing	292,941 (12)
**Neonatal intermediate care (2000–2009)**	
Yes	84,3713 (36)
No	1,231,501(52)
Missing	292,941 (12)
**Demographic composition**	Median (Q1, Q3)
Proportion Black infants	0.09 (0.03,0.21)
Proportion Medicaid-insured infants	0.43 (0.25, 0.59)
Proportion lowest income ZIP code quartile residents	0.16 (0.03, 0.39)

**Table 3:** Adjusted odds of BPBI by infant and intrapartum characteristics

Adjusted Odds of BPBI by Infant and Intrapartum factors				
	AOR	95% CI		p value
**Male**	0.81	0.78	0.84	<0.0001
**Race/Ethnicity**				
Black race	1.62	1.52	1.72	<0.0001
Hispanic ethnicity	1.29	1.22	1.37	<0.0001
Asian/Pacific Islander	0.93	0.84	1.02	0.1331
Native American	1.06	0.84	1.34	0.6160
Other	1.30	1.19	1.42	<0.0001
Unknown	0.97	0.89	1.06	0.4966
White	Reference			
**Insurance**				
Medicaid	1.14	1.09	1.20	<0.0001
Other	0.99	0.90	1.08	0.8002
Private	Reference			
**Lowest quartile of ZIP-code median income**	1.08	1.03	1.14	0.0027
**Intrapartum factors**				
Shoulder dystocia	65.28	61.38	69.42	<0.0001
Macrosomia	2.82	2.60	3.05	<0.0001
Vaginal delivery	7.06	6.53	7.63	<0.0001
Multiple gestation	0.63	0.51	0.77	<0.0001
>41 weeks gestational age	3.86	3.67	4.06	<0.0001

**AOR** (Adjusted Odds Ratios) were each estimated in a multiple logistic regression model simultaneously controlling for the other factors in this table as well as year of birth and the hospital factors in table 4

**Table 4:** Adjusted odds of BPBI by hospital characteristics

Adjusted Odds of BPBI by Hospital Characteristics				
	AOR	95% CI		p value
**Delivery Volume**				
10–500 births	1.04	0.94	1.14	0.4917
501–1000 births	0.99	0.90	1.09	0.8307
1001–2000 births	0.97	0.88	1.07	0.4994
>2000 births	Reference			
**Teaching status/location**				
Rural	0.90	0.82	0.997	0.0426
Urban, Non-teaching	0.95	0.90	1.01	0.0914
Urban, Teaching	Reference			
**Ownership**				
Public (government, non-federal)	1.10	1.03	1.17	0.0027
Private, for profit	0.83	0.77	0.89	<0.0001
Other	1.01	0.83	1.23	0.9146
Private, not-for-profit	Reference			
**AHA Obstetric Care Level**				
Level 1 (uncomplicated)	1.19	1.03	1.39	0.0215
Level 2 (uncomplicated, many complicated)	1.16	1.03	1.30	0.0152
Level 3 (all serious illnesses and abnormalities)	Reference			
**Neonatal Intensive Care**	1.04	0.93	1.16	0.4657
**Neonatal Intermediate Care**	0.97	0.87	1.07	0.5077
**modified Unexpected Complications in Term Newborn index (for a 1 IQR increment)**	1.03	1.00	1.06	0.0243
**Demographic composition**				
Highest vs Lowest quartile: Black births	1.35	1.22	1.48	<0.0001
Highest vs Lowest quartile: Medicaid births	0.95	0.86	1.04	0.2367
Highest vs Lowest quartile: Low Income births	1.01	0.92	1.10	0.8579

**AOR** (Adjusted Odds Ratios) were each estimated in a multiple logistic regression model simultaneously controlling for the other factors in this table as well as year of birth, infant characteristics, and known intrapartum factors (shown in Table 3).
